# A Review of Dementia Caregiver Interventions: Valuing Psychological Well-Being and Economic Impact Through the State-Preference Method

**DOI:** 10.3390/ijerph23010104

**Published:** 2026-01-12

**Authors:** Anna Consiglio, Antonella Lopez, Andrea Bosco

**Affiliations:** 1 Department of Educational Sciences, Psychology and Communication, University of Bari, 70122 Bari, Italy; anna.consiglio@uniba.it (A.C.); andrea.bosco@uniba.it (A.B.); 2 Department of Humanities, Social Sciences and Education, University of Molise, 86100 Campobasso, Italy

**Keywords:** caregiver burden, psychological intervention, WTP/WTA, psychometrics

## Abstract

**Highlights:**

**Public health relevance—How does this work relate to a public health issue?**

- Caregiver-focused psychological interventions reduce distress and burden; economic evidence is growing but often methodologically heterogeneous.

**Public health significance—Why is this work of significance to public health?**

- A PRISMA-based synthesis linking clinical outcomes with costs and stated-preference evidence (WTP/WTA), showing robust value signals for START and TAP, mixed utility gains for online CBT, and limited effects for ATT.

**Public health implications—What are the key implications or messages for practitioners, policymakers and/or researchers in public health?**

- Standardizing utility measurement and WTP/WTA elicitation—and adopting societal perspectives that include informal care—will strengthen value assessments and guide scalable caregiver support policies.

**Abstract:**

Objectives. To integrate clinical and economic evidence on the main non-pharmacological interventions aimed to reduce the burden of caregivers of people with dementia, with specific attention to stated preference measures (SPM), Willingness-to-Pay (WTP) and Willingness-to-Accept (WTA), alongside other cost-effectiveness indicators (ICER, QALY). Methods. A systematic review was conducted on randomized and quasi-experimental evaluations, economic models, and preference studies concerning psychoeducational/coping interventions, activity-centered/occupational programs (TAP), technological solutions and tele-support, and goal-oriented cognitive rehabilitation (CR). For each study, the following indexes were extracted: design, sample size, psychological outcomes (anxiety/depression, burden, engagement), utility per QALY, costs per perspective (the health–social and the broader societal perspectives), ICER, WTP/WTA, and sensitivity results. Results. Psychoeducational programs and CR show consistent benefits on distress, anxiety/depression, and caregiver quality of life; TAP reduces caregiver burden and patient behavioral problems, with favorable signs of cost–effectiveness; results on the effects of technologies are heterogeneous, but online modules with telephone support improve psychological morbidity. QALY improvement is generally modest, but the probability of cost-effectiveness remains high when costs do not differ significantly from treatment as usual, or when, from a societal perspective, the unpaid caregiving time of the caregiver is valued. Preference studies indicate positive WTP for additional hours of home care, health–social integration, and facilitated groups; evidence on WTA is scarcer and methodologically variable. Conclusions. Short, structured interventions with a human support component offer good value-for-money; the adoption of societal perspectives and the systematic use of WTP/WTA can better capture the value perceived by caregivers. Heterogeneity issues persist.

## 1. Introduction

### 1.1. Introduction

With an estimated 78 million cases worldwide in 2030 and 139 million in 2050, dementia is one of the most widespread and challenging pathological conditions globally [1]. The impact of the state of pervasive disability that dementia implies, directly proportional to the severity of the condition, appears to be overwhelming for the individuals living with dementia, as well as for their caregivers, families, and society at large.

Informal carers play a vital role in the provision of a dignified life for people struggling with the disease [2], in terms of physical and emotional care. Family carers are often referred to as the invisible second patient, as the impact of being a carer also implies high rates of distress and psychological morbidity, as well as social isolation, physical illness and financial hardship [3]. Unlike formal caregivers (e.g., care manager), the large majority of informal carers have no prior experience with providing medical, nursing or psychological cares: most family caregivers lack the time to prepare themselves for their care role [4] and this leads to the establishment of an overload or burden.

The concept of caregiver burden encompasses a range of negative impacts experienced by individuals assuming the primary caregiving role, and it is generally defined as the degree to which the caregiver’s physical and mental health, social life and economic status enter a state of distress because of the care activity [5]. These impacts can manifest as both subjective feelings and objective consequences [6]. Objective caregiver burden quantifies the practical aspects of caregiving, such as the time invested and specific tasks performed. In contrast, subjective burden reflects the caregiver’s personal experience in performing the task of caregiving, encompassing the physical, psychological, emotional, social, and financial impacts that they endure in a multifaceted way [7]. Moreover, the economic strain impacts psychological well-being in terms of worrying about finances, financial constraints and productivity loss, which may limit their ability to participate in social activities and can make caregivers more vulnerable to physical and mental health problems, and, in turn, impact their ability to provide care [8].

Support to caregivers can be provided in various forms, including instrumental (assistance with daily living activities and household tasks), financial, medical, emotional, and educational care [9,10,11,12], as reported in the meta-review of Cheng and Zhang [13], which focuses on the psychological promotion of well-being and is limited to non-pharmacological interventions and support. The non-pharmacological interventions for informal dementia caregivers are broadly categorized into two main areas: (a) psychosocial interventions for caregivers (which include psychoeducation, counseling, psychotherapy, supervised support groups, and behavioral management) which enable the caregiver to carry out their caring duties with increased support and resources; social service and respite care (i.e., a temporary rest time from caregiving) while the person living with dementia continues to receive professional care in a safe environment [14] are also included; (b) interventions involving both patients and caregivers (including dyadic reminiscence, exercise programs, occupational therapy, cognition-oriented therapy and care coordination) intended to improve the quality of the time spent together by the caregiver and the patient. 

The evaluation of the effectiveness of psychological interventions for informal caregivers of people with dementia has typically relied on multidimensional outcomes, including caregiver burden, depressive symptoms, anxiety, and overall quality of life [15]. Evidence suggests that psychoeducational and cognitive–behavioral approaches can reduce psychological distress and improve coping strategies, although the strength and duration of these effects vary [16]. More recent systematic reviews highlight that interventions tailored to caregivers’ specific needs, delivered with sufficient intensity and follow-up, tend to yield more robust benefits [17]. However, heterogeneity in study designs, outcome measures, and follow-up duration complicate the comparability of findings, underscoring the need for standardized methodologies to better assess post-intervention effectiveness in this population [18].

### 1.2. Economic Value of Dementia Care

The cost of caregiving has been viewed as one of the most important agendas in health and social care [1,19]. While healthcare costs represent a substantial component of the overall burden of dementia, they are typically outweighed by the financial and resource demands associated with social care and informal caregiving services provided within the community [20]. The financial implications of informal caregiving have thus become a prominent area of investigation in recent years [21], given its substantial influence on familial structures and well-being and the broader economic burden associated with numerous chronic conditions. The underlying issue is to provide an analysis considering all the relevant components of costs, then including direct (expenditures incurred by the healthcare system for dementia diagnosis, treatment, and prevention), indirect (the value of resources lost due to the disease, including premature mortality, reduced productivity of both patients and caregivers), and intangible costs related to caregiving—such as the pain of losing a loved one or the pain of watching a loved one suffering because of disease or going through a tough course of treatment [22]. Psychological research should prioritize the study of these intangible costs to give full recognition to the marginalized dimension of caregiving, and also take advantage of the information that comes from studies carried out in the economic field.

As the scientific literature suggests [23,24], among the techniques of economic evaluation in healthcare, Cost–Utility Analysis (CUA), Cost–Benefit analysis (CBA) and Cost-Effectiveness Analysis (CEA) are the most common measures applied in interventions, and could also be useful in the field of non-pharmacological and psychological interventions. Studies have thus investigated the impact of incorporating the cost of informal care on the outcomes of cost-effectiveness studies. They can strongly influence the probability of whether interventions are deemed cost-effective or not [25]. In our view, the integration of economic evaluations in the design and assessment of dementia-related interventions falls perfectly within both the purposes of quantitative psychology and the spirit of interdisciplinary collaboration between psychologists, health economists, and public health experts [26].

### 1.3. The Present Study

Although both the economic impact and psychological effects of caregiving have been acknowledged, few studies have systematically integrated these two dimensions, particularly in the evaluation of non-pharmacological interventions for dementia caregivers. This scarcity is compounded by a limited history of economic evaluation in psychological care, resulting in a lack of systematic cost-effectiveness assessments [27].

The present study aims, as a first objective, to conduct a review, targeting caregivers of patients with dementia, concerning psychological interventions that incorporate cost evaluations alongside assessments of efficacy, to ascertain the extent to which this practice is prevalent within the field. This approach may hold significance both in terms of generating data that is useful for the development and refinement of healthcare policies and guidelines, and in terms of supporting the implementation and strategic orientation of non-pharmacological interventions as cost-effective or value-for-money solutions. 

Considering the growing interest among international regulatory agencies in integrating top–down and bottom–up approaches, even in dementia burden care, this review focuses on studies utilizing stated-preference measures like Willingness-to-Pay (WTP) and Willingness-to-Accept (WTA). These measures have been chosen for their practical relevance as well as because they are inherently tied to the psychological discipline, directly addressing individuals’ perspectives and involving complex decision-making processes. For instance, the endowment effect [28] serves as a key example of the deep connection between this methodology and the psychological mechanisms of decision-making, highlighting how individuals place a disproportionately high value on their possessions. We will explore how these methods and their findings can offer practical utility to stakeholders, both in empirical research and in the design of tailored interventions for caregivers of individuals with dementia. The focus on WTP and WTA is not merely a methodological choice but represents the primary distinguishing filter of this review. This emphasis is absolute because these measures move beyond traditional, objective cost-effectiveness analysis to directly capture the subjective value and psychological cost of caregiving. Specifically, WTP/WTA studies are unique in that they force the caregiver to express the trade-offs they are willing to make—in terms of money, time, or emotional strain—to avoid or mitigate the burden. This valuation process is deeply linked with psychological constructs such as loss aversion (as exemplified by the endowment effect), risk perception, and the perceived utility of relief. By focusing on WTP and WTA, we aim to review the literature that inherently links the financial dimension with the psychometric perspective of the individual, providing a bottom–up economic signal essential for true patient-centered resource allocation.

However, it must be noted that a comprehensive review addressing, in part, this subject has already been published in the literature. A recent example of integrated cost-effectiveness analysis in interventions for caregivers of people with dementia is the systematic review by Huo and colleagues [29], which examined 33 studies on caregiver psychological health, burden, and quality of life. While unquestionably valuable, it did not include stated-preference measures, which are central to our approach.

Evaluating intervention effectiveness is inherently part of the measurement framework and standard practice in clinical settings, especially in mental health, where objective biomarkers remain limited [30]. For this reason, intervention effectiveness and cost assessment are also considered important in this study. Moreover, quantitative methodologies, such as in econometrics and biostatistics, have increasingly become part of quantitative psychology and psychometric practice, reflecting the transferable nature of scientific measurement [31]. Integrating economic and psychometric perspectives enables a more comprehensive understanding of caregiving burden, supports better intervention design, and generates evidence to guide policy and resource planning.

## 2. Materials and Methods

This paper is structured in accordance with the Preferred Reporting Items for Systematic Reviews and Meta-Analysis (PRISMA) guidelines [32]. No registration information is required. 

### 2.1. Search Strategy

An extensive literature search was conducted using electronic databases, using the SCOPUS database. The search terms used in all these searches were organized into four blocks including (i) caregiver burden; (ii) intervention (promotion of mental health and well-being); (iii) economic evaluation (e.g., CEA, CUA, WTP, WTA); and (iv) dementia as the disorder affecting the person in need of care and supported by the caregiver; no year limits were set. No limits regarding the publication year were set. This phase of study selection was completed on 1 December 2025. All citations were imported into an electronic spreadsheet in which the duplications were eliminated. After the first step of selection on Scopus, we checked for possible further publication records on Pubmed Central, which did not yield any additional entries. 

### 2.2. Inclusion and Exclusion Criteria

English peer-reviewed articles published in scientific journals were considered eligible; gray literature was not considered. Exclusion criteria were applied to (a) conference papers, if not available in their full-text versions and/or if the reported contents had also been published in the form of an article; (b) conference reviews; (c) book chapters, when full texts were not available; (d) notes; (e) books; and (f) retracted papers. The subject area was a further selection criterion of the selected literature; they were published in journals classified in the areas of psychology, nursing and medicine. The primary focus of this review was on the economic and effective evaluation of interventions targeting informal caregivers of individuals with dementia, as well as dyads composed of the caregiver and the patient. In mainly considering the viewpoint of health economics, studies that presented interventions aimed at informal caregivers or dyads of informal caregivers / family members with dementia were retrieved and identified. Papers on patient-only interventions that documented positive effects on caregivers at home were also considered. Furthermore, as the subject of this study, it was deemed inappropriate to include other clinical conditions with cognitive impairment and/or chronic psychiatric or physical pathologies in the study. Table 1 shows the syntax of the search query used in the Scopus database.

### 2.3. Data Extraction and Article Selection

The records selected were screened for eligibility in two further steps. First, they were assessed by title, abstract, and keywords, to make sure that they met all the exclusion and inclusion criteria. Identified eligible records were extracted and their full texts were downloaded. As a final check, the last step for eligibility consisted of reading the full texts and checking for the inclusion/exclusion criteria. The process of article selection was conducted by two independent reviewers (AC and AL). A total of 14 articles were included in this systematic review. 

**Figure 1:**
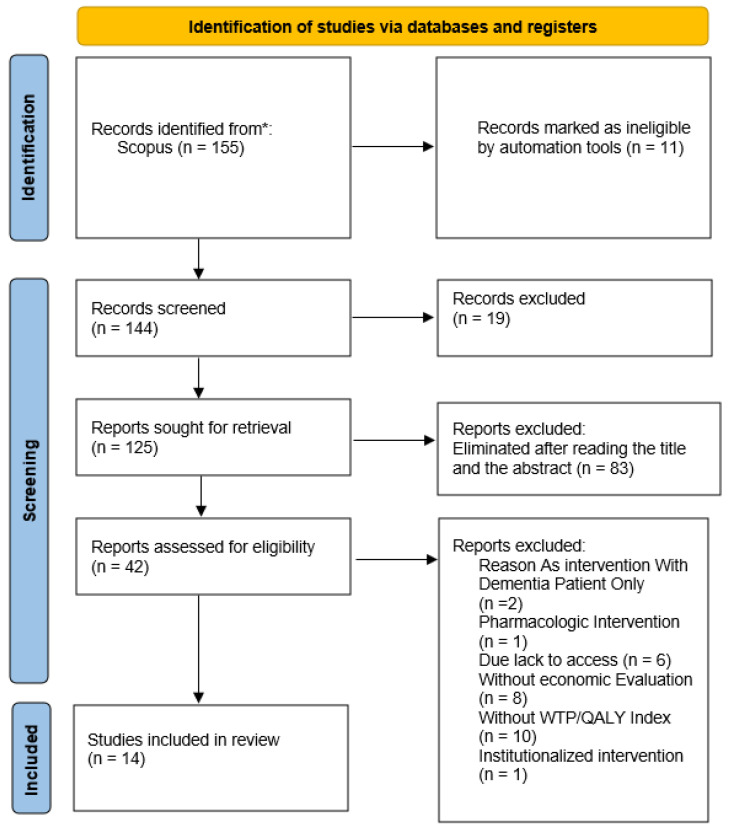
Systematic review flow chart detailing the literature search, number of abstracts screened, and full texts retrieved. *: SCOPUS database.

At the end of the methodological process, a total of 14 articles were included in this systematic review. The entire process of the search strategy and article selection is displayed in Figure 1.

### 2.4. Risk-of-Bias Assessment

In consideration of the substantial heterogeneity of the study designs included in this review, which range from randomized controlled trials to economic modeling and stated-preference studies, a formal risk-of-bias assessment using standardized tools (e.g., RoB 2, ROBINS-I) was not deemed to be useful. Methodological guidance acknowledges that such tools may have limited applicability in reviews integrating heterogeneous clinical and economic evidence [32,33].

Instead, a narrative assessment of methodological quality was conducted, in accordance with established recommendations for narrative synthesis in systematic reviews [34]. The classification of studies was conducted through a qualitative analysis, which entailed the assessment of risk of bias. The following aspects were considered in the study design and internal validity: (i) the clarity and replicability of the intervention; (ii) the appropriateness and validity of psychological outcome measures; (iii) the transparency and plausibility of economic assumptions, including stated-preference elicitation methods; and (iv) the completeness of reporting.

This methodological approach permitted a context-sensitive evaluation of its limitations while preserving the comparability of heterogeneous study types, in accordance with the PRISMA 2020 reporting principles [32].

The review was not registered in PROSPERO, as it integrates economic preference elicitation studies and modeling approaches that are not consistently accommodated within existing registration frameworks.

### 2.5. Quality Assessment Checklist

The set of selected articles mainly includes studies with experimental and quasi-experimental research designs, but also randomized controlled trials, and interventions and metanalysis. The checklist was inspired by that used by Spano and colleagues (2023) and based on two available checklists from the Joanna Briggs Institute’s critical appraisal tools for use in JBI Systematic [35], that is, the Checklist for Quasi-Experimental Studies (Non-Randomized Experimental Studies) and the Checklist for Randomized Controlled Trials. The present checklist consisted of eight items scored on (Q1) research design (What type of research design was applied?); (Q2) control group (Was there a control group for the main outcomes?); (Q3) comparability between samples (Were participants included in any comparisons similar in terms of demographics?); (Q4) perspective (Is the actual perspective chosen appropriate?); (Q5) measures (Were outcomes measured in a reliable way (validated scales)?); (Q6) statistical analysis (Was appropriate statistical analysis used?); (Q7) appropriateness of results (Were all the mentioned outcomes appropriately reported in the result section?); and (Q8) presence of power analysis for sample size calculation (Was a power analysis conducted to establish sample size?). Each item was scored with 1 (Yes/addressed/clear), 0.5 (Partially addressed/partially clear), or 0 (No/not addressed/not clear). Quality assessment was conducted by two independent reviewers (AC and AL). The interrater reliability as assessed with the Cohen’s Kappa coefficient for the eight items of the quality assessment has reached the perfect agreement 1 (*p* < 0.001). The total score of the quality assessment checklist was obtained by adding up the scores of each item of the checklist, but only for articles. Table 2 reported the selected studies as classified into four categories: “low quality” (score < 3), "medium quality” (3 ≤ score < 5), “high quality” (5 ≤ score < 7) “very high quality” (score ≥ 7). Thirteen studies were classified as having a very high quality and one study as having high quality. The lowest average scores are associated with the (Q1) research design and the control group, (Q3) comparability between samples, (Q4) perspective, (Q5) measures, (Q6) statistical analysis, (Q7) appropriateness of results, and (Q8) presence of power analysis for sample size calculation research design, item measured in a reliable way and the presence of a control group. On the other hand, all the included studies and the study results were described unambiguously and completely.

## 3. Results

### 3.1. Characteristics of Included Studies

As stated before, the fourteen articles included stated-preference measures. In order to classify the articles, we chose to adopt the categorization introduced by Huo and colleagues [29] in their literature review (whose criteria are summarized in Supplementary File S1). They [29] propose a classification of caregiver interventions based on the existing studies. This decision is justified by the fact that the cited review assesses the effectiveness of interventions and includes an evaluation of their economic costs as well, making it more aligned with the objectives of our research, even if it is not focused on the state preference method approach.

Where possible, costs were converted into euros and updated using the average inflation rate for 2025. With respect to cost evaluation approaches and methodologies, considering the main different approaches of economic analytic designs in the current literature, it was found that the selected paper mentioned in Table A1 and Table A2 conducted economic evaluations by adopting the Contingent Evaluation Method (CVM) and the Cost-Effectiveness Analysis (CEA). In only one case [36], the WTP values were carried out using the Discrete Choice Experiment (DCE) as a State Preference (SPM) technique. 

Regarding the type of intervention approaches, two proposals refer to telecare [37,38], one of which refers to managing the transition between institutionalization/hospitalization and returning home [38]. In general, these are personalized approaches, often involving the dyads. However, all the proposed intervention programs are non-pharmacological and tendentially multicomponential (psychological, training/support and education/support). Moreover, the Randomized Control Trials (RCT) protocol is mostly used [38,39,40,41,42].

Overall, most randomized controlled trials and structured psychosocial interventions had a low-to-moderate risk of bias. Studies based on stated preference elicitation and economic modeling had a higher risk of bias because of hypothetical valuation, context and modeling assumptions.

Strategies for Relatives (START) is a psychoeducational intervention for informal caregivers of people with dementia. Delivered by trained professionals, it focuses on enhancing caregivers’ coping strategies, stress management and psychological resilience using cognitive–behavioral techniques. By improving emotional regulation, problem-solving skills and responses to behavioral and psychological symptoms of dementia, START modifies interactional patterns within the dyad, thereby contributing to improved relational quality and more adaptive caregiving practices.

The Tailored Activity Program (TAP) is a program for people with dementia and their caregivers. It involves activities designed to enhance the well-being of the person with dementia and reduce the caregiver’s burden. TAP is based on the cognitive abilities, interests, and environmental context of each person. It also trains caregivers to implement and adapt these activities. This improves daily interactions within the dyad, reducing behavioral disturbances and increasing shared positive experiences. TAP also reduces the time, stress, and emotional load associated with caregiving.

ATTILA RCT (ATT) intervention is a echnology-based solution aimed at enhancing safety and supporting independent living for people with dementia. These interventions, which include remote monitoring systems, telecare services and digital support platforms, alter patterns of dependence and supervision, potentially increasing autonomy while preserving caregiver well-being. For caregivers, ATT interventions reduce vigilance demands, uncertainty and perceived responsibility, thereby alleviating psychological strain.

### 3.2. Research Findings

To facilitate interpretation and ensure clarity in reporting, the results of the review have been summarized in Table A1 and Table A2.

Table A1 presents interventions and state preference method experiments along with category, description and goals, duration, human resources required, cost of intervention, cost split per patient and caregiver, intervention effectiveness, Willingness-to-Pay (WTP), and Willingness-to-Accept (WTA). In Table A2, for each included study, the following data were extracted and reported: the Incremental Cost-Effectiveness Ratio (ICER), Cost–Utility, Quality-Adjusted Life Years (QALY), Disability-Adjusted Life Years (DALY), WTP, WTA, and sensitivity analysis results. A detailed description of each of these indices is provided in Supplementary File S2. This detailed extraction of indicators was undertaken to ensure a comprehensive comparison of studies and outcomes, thereby facilitating an integrated evaluation of the interventions across multiple economic and clinical dimensions.

Table A2 shows heterogeneity with regard to the structure of the programs. Overall, there are individual 1:1 sessions and small group therapies; the duration ranges from three to six months (with follow-up up to nine months), and the number of sessions varies as well, from 8 to 15. The sample sizes vary from a few dozens to several hundreds of people, with the target group being caregivers only or dementia patients (with mixed clinical conditions and different severity) and their caregivers. The number of professional resources involved, and the composition of the teams vary: teams can be composed of psychologists, occupational therapists, nurses and other specialized personnel.

Across the reviewed studies, structured psychosocial and psychoeducational interventions—particularly those based on the START framework—were associated with consistent improvements in caregiver psychological outcomes. From an economic perspective, Willingness-to-Pay estimates indicated that caregivers assign positive monetary value to interventions that enhance coping strategies, emotional support, and care coordination. However, Willingness-to-Accept measures were rarely employed, which limited the assessment of perceived losses associated with caregiving burden and constrained the interpretability of economic trade-offs. The findings of this study constitute a narrative synthesis of heterogeneous studies, as opposed to pooled quantitative estimates, and should, therefore, be interpreted with appropriate caution.

### 3.3. Stated Preference Methods

Nine studies used stated-preference methods to measure the value that caregivers and, in some cases, people with dementia, place on non-pharmacological interventions. Overall, these studies demonstrate that Willingness-to-Pay (WTP) increases with the intensity and personalisation of interventions, as well as the perceived reliability of services. Meanwhile, Willingness-to-Accept (WTA) captures the perceived opportunity costs and burden associated with caregiving. Valuations are consistently influenced by caregiver characteristics (e.g., income, gender and relationship to the care recipient) and care recipient characteristics (e.g., cognitive status, disability severity and care needs).

Across both types of experiments, personalized and in-home interventions elicited higher WTP, particularly when interventions were tailored to the functional and cognitive profile of the person with dementia. Time losses associated with caregiving were often valued at levels comparable to paid care work, indicating the substantial social and economic costs borne by informal caregivers [50]. Studies that distinguished between unilateral and altruistic preferences revealed that caregivers and patients may assign different values depending on whether the benefits are perceived as individual or shared within the dyad [51,52].

However, a recurring finding was the substantial proportion of non-responses—up to one third of caregivers—particularly for WTA measures. This suggests cultural, ethical, or personal resistance to monetising the caregiving experience [50]. Discrete Choice experiments also revealed strong preferences for integrated home care services that combine health and social support, professional supervision and continuity of care. These preferences vary according to the characteristics of the caregiving dyad, indicating the need for flexible, personalized caregiving policies [36].

### 3.4. Clinical Effectiveness

A general trend of significant improvements in caregivers’ mental health was observed across the reviewed interventions, particularly in terms of reductions in anxiety and depression [38,39,40,43]. Improvements in caregivers’ quality of life were reported less consistently, but were observed in some studies. Most interventions were evaluated over short-to-medium-term follow-up periods (6–18 months), which limited conclusions regarding long-term sustainability [38,43]

Among the interventions, the psychological training program START was found to be the most methodologically robust. Extended follow-up analyses confirmed sustained reductions in caregiver anxiety and depression, together with quality of life improvements in both the short- and long-term [39,40]

Tailored activity-based programs, including TAP, demonstrated reductions in caregiving tasks and behavioral disturbances. This indicates that the benefits are primarily related to daily functioning and interaction patterns within the caregiver–care recipient dyad [44].

Assistive technology and telecare interventions showed more limited and inconsistent effects, with minor reductions in caregiver burden generally observed [37].

Other approaches, such as online cognitive–behavioral therapy (CBT) and goal-oriented cognitive rehabilitation, produced mixed results: online CBT was associated with reductions in anxiety and depression, as well as modest quality-of-life improvements [38], while cognitive rehabilitation produced larger post-intervention and maintenance effects [43].

## 4. Discussion

The findings of the present study may contribute to the evaluation of clinical interventions and the cost-effectiveness of non-pharmacological interventions for caregivers of people with dementia, with reference to the stated-preference approach, which tries to combine quantitative psychology/psychometrics and economic point of views. Results show that psychosocial, technological, and activity-based interventions, and dementia management programs were subjected to combined evaluations of clinical effectiveness and economic outcomes. Variability in study design and the absence of standardized protocols limited comparability across studies. Rather than providing pooled quantitative estimates, the findings should be interpreted as a more precise narrative synthesis of heterogeneous evidence, reflecting differences in intervention content, study design, outcome measures and economic frameworks. 

### 4.1. Types of Interventions and Clinical Effectiveness Evaluations

From a clinical perspective, psychosocial, activity-based and technology-supported interventions demonstrate different patterns of effectiveness. Interventions such as START demonstrate sustained reductions in caregiver anxiety and depression, as well as favorable cost-effectiveness profiles across multiple thresholds, suggesting a relatively high degree of transferability to real-world settings [39,45]. Activity-based interventions, including TAP, appear promising in reducing caregiver burden and delaying institutionalization, indicating potential clinical and organizational benefits [51,53]. Cognitive rehabilitation for individuals with early-stage dementia has been shown to improve functional and psychosocial outcomes, highlighting its potential relevance for specific caregiver–patient dyads [50]. In contrast, assistive technology and telecare interventions have more limited and inconsistent effects on caregiver outcomes. This suggests that technological solutions alone may be insufficient if they are not embedded within structured support models [37] CR reduces anxiety and depression and improves quality of life to a modest extent, highlighting the importance of human support components in digital interventions [38].

### 4.2. Types of Interventions and Cost Evaluations

When aligned with recommendations from national/international regulatory bodies, including WHO, NICE, and Guiding an Improved Dementia Experiences—USA (CSM), some non-pharmacological interventions, particularly TAP and START, demonstrated, comparatively, cost-effectiveness and consistency with recommended caregiver support practices. TAP, an occupational therapy-based intervention designed to engage individuals with dementia in structured, meaningful activities and train caregivers to integrate tailored, daily activities, demonstrated cost-effectiveness in reducing caregiver burden and delaying institutionalization [45,51]. Despite general policy support for assistive technologies by WHO and NICE, the ATT intervention is not currently recommended due to insufficient evidence for economic effectiveness [52].

### 4.3. Clinical Effectiveness and Cost-Effectiveness of Key Caregiver Interventions

When clinical outcomes are considered alongside economic evidence, only a subset of interventions—most notably START, and to a lesser extent TAP—show a relatively coherent alignment between validated psychological outcomes and standardized economic indicators, such as QALYs and ICERs [39,40,46]. In contrast, other interventions show discrepancies between subjective caregiver benefits and utility-based economic outcomes. Notably, interventions such as online CBT can provide significant psychological relief while yielding modest QALY gains and relatively high ICERs. This divergence highlights the important role of psychological processes, such as perceived control, coping and acceptance, in determining the subjective value of caregiving interventions. While QALYs remain essential for resource allocation and policy decisions, they may underestimate benefits that are primarily experiential or relational in nature.

Importantly, modest QALY gains—in our opinion—should not be considered trivial from a policy perspective. In the context of dementia caregiving, for example, even small improvements in the quality of life of caregivers can translate into substantial population-level benefits when interventions are scaled up and sustained over time, particularly through delayed institutionalization and reduced healthcare utilization. These effects are particularly relevant in aging societies where informal caregiving is a significant, yet frequently overlooked, part of long-term care systems.

Analysis of Stated-preference Studies Further Highlights the Potential and Current Limitations of WTP and WTA Approaches.

While these methods capture dimensions of value that extend beyond utility-based metrics, such as time costs, perceived burden and relational benefits, their application remains methodologically fragmented. Inconsistencies in cost definitions, outcome measures, ICER calculation methods and preference elicitation techniques limit comparability across studies. Furthermore, WTA measures are rarely reported, despite their theoretical relevance for capturing perceived losses associated with caregiving. These gaps constrain the full integration of stated-preference evidence into decision-making processes.

More in depth, from a methodological standpoint, preference values are derived using diverse instruments and valuation frameworks, influenced by national economic contexts and population characteristics. Although several studies adopt theoretical models incorporating spillover effects and family altruism [48,49], few evaluations systematically integrate these dimensions into economic analyses. The choice of utility instruments further affects estimated ICERs, and direct comparisons between psychometric outcomes and QALYs remain limited. Together, these issues highlight the need for greater methodological harmonization in future research.

Data analysis of state preference experiments revealed gaps in cost per patient/target variables, differences in ICER calculations (QALY-based vs. test-based), experimental WTP thresholds (except NICE, which has its own threshold per policy), and WTA preferences, all of which are not sufficiently present. To address such gaps, both service design and research protocols should systematically include validated psychological instruments to detect potential improvements in participants, aligned with the key features of the chosen intervention approach (e.g., reducing caregiver burden; improving mood (depression/anxiety); reducing stress, teaching coping strategies), alongside harmonized economic metrics (e.g., QALY, DALY, ICER). This would ensure a multidimensional understanding of intervention outcomes that align clinical relevance with health–economic sustainability.

### 4.4. Discrepancies Between Subjective (WTP/WTA) and Top–Down Economic Estimates

The comparison between bottom–up, individual, behavioral assessments (WTP/WTA), and top–down institutional cost estimates [1] suggests a substantial mismatch between caregivers’ subjective estimations and formal health expenditure accounting. This divergence underscores the systematic undervaluation of caregiving time in subjective appraisals, emphasizing the need to formally recognize caregiving as a component of indirect healthcare costs in institutional frameworks. Moreover, in top–down datasets, estimates of hours saved are inferred from monetary benefits, not from a direct measurement of time use. Regional averages may overlook intra-regional socioeconomic differences and potential indirect and long-term health system savings (e.g., delayed institutionalization) are not captured. The cost of implementation (training, supervision, digital infrastructure) is not fully integrated into unit cost analysis.

### 4.5. Psychological Efficacy and the Role of Clinical Health Psychology

Several of the interventions reviewed are firmly grounded in clinical and health psychology, in terms of both theoretical framework and professional delivery. The START program is a worthy example, involving psychology graduates supervised by licensed clinicians, and demonstrating robust reductions in depression and anxiety as well as long-term economic benefits. Activity-based interventions (e.g., TAP) contribute to improved patient–caregiver interactions and daily functioning, although they are often evaluated without standardized psychometric tools. These findings affirm the critical role of clinical psychology in dementia care: programs led or supervised by psychologists not only improve caregiver well-being but also yield psychometrical measurable savings in healthcare and social costs. The establishment of a multidisciplinary team—comprising clinical psychologists, psychometricians, and health economists—is strongly recommended to ensure comprehensive intervention design, implementation, and evaluation [49,54].

### 4.6. Methodological Considerations in Economic Evaluations

Regarding methodological aspects, the data reveal a lack of standardization in economic preference measures across studies. state preference values (e.g., WTP/WTA) are defined independently within each study, using different instruments (e.g., payment cards, surveys, etc.) contributing to substantial variability in estimates due to differing valuation methods, population characteristics, and national economic contexts. The substantial methodological heterogeneity observed across studies significantly limits both their comparability and the generalizability of findings. From a theoretical perspective, economic studies are framed in models that consider spillover effects, i.e., the inclusion of indirect costs and caregiver effects, and in approaches based on family altruism, where patient and caregiver preferences are interdependent. Furthermore, many analyses fit into the paradigm of cost–benefit analysis in contexts without market prices or different socioeconomic backgrounds [47]. However, several methodological gaps emerge: a few evaluations systematically integrate spillover effects and preference reciprocity, limiting the full estimation of the social value of interventions. Furthermore, the choice of utility measurement instruments can significantly affect utility values and ICERs. Finally, the lack of direct comparison between traditional outcomes and QALYs is highlighted, as well as the need to standardize the use of a single state preference elicitation method to ensure comparability between health policies, from a societal perspective. 

Furthermore, some limitations emerged: (a) the lack of standardized economic evaluation: few studies utilize stated preference methods (e.g., those often combined with QALYs) or standardized economic tools, severely limiting cross-study comparison; (b) divergence in valuation: data derived from caregiver reports (bottom–up) often underestimate the true economic burden compared to institutional estimates (top–down); (c) measurement bias: economic benefits from reduced caregiver time are typically inferred from cost proxies rather than being directly observed via time-use data; (d) methodological heterogeneity: variability in outcome measures, time horizons, and costing perspectives limits the generalizability and robustness of findings; and (e) implementation costs are frequently not fully included, potentially impacting scalability in practice. These limitations underscore the urgent need for harmonized multidimensional evaluation frameworks that integrate clinical, psychological, and economic metrics.

## 5. Conclusions

This review underscores the growing importance of non-pharmacological interventions in supporting caregivers of people with dementia—both as a public health priority and as a cost-effective complement to clinical care. However, it becomes apparent that studies evaluating the cost-effectiveness of interventions targeting caregivers of individuals with dementia including state preference tools are still relatively scarce; in a broader scientific production context where limited attention is often paid to the joint evaluation of cost and effectiveness, it is essential to recognize that—despite varying public policy frameworks and healthcare coverage systems across geographic regions—economic sustainability remains a critical prerequisite for the real-world scalability of any scientifically validated intervention. The evidence confirms that when properly targeted and scaled, these programs not only may improve caregiver well-being but also generate substantial societal savings through reduced informal care demands and potential delays in institutionalization. While differences in cost structures, delivery infrastructure, and caregiver demographics necessitate region-specific strategies, countries with structured community healthcare systems, such as Italy, are particularly well-positioned to integrate these interventions into routine practice. Nevertheless, the variability in methodological approaches and the scarcity of studies using preference-based outcomes point to a need for more rigorous, harmonized evaluations. 

Future research should prioritize standardized economic metrics and psychometrics, incorporate caregiver spillover effects, and assess long-term impacts on quality-adjusted life years (QALYs) and healthcare utilization. The state preference method could improve the feasibility of study and choice of cost-effective and sustainable intervention methods, with significant implications for the accessibility and acceptability of the target population in a specific area. Overall, this work reinforces the case for embedding evidence-based, scalable caregiver support programs within national dementia strategies, particularly in the face of aging populations and increasing informal care burdens.

### 5.1. Limitations

The cost-effectiveness research focusing specifically on caregivers of persons with dementia remains limited, particularly those adopting state preference methods (e.g., those often combined with QALY-based assessments). Many interventions are either not evaluated using standardized economic tools or rely on indirect estimates, making cross-study comparisons challenging. Second, while the present work integrates both bottom–up and top–down data (e.g., Willingness-to-Pay estimates and WHO/EU cost benchmarks), these approaches often yield divergent results. However, in our view, this aspect should not be regarded as a limitation, but rather as an important implication to consider—one that is inherent to the approach required for chronic and pervasive conditions such as dementia, where the social dimensions of the illness are particularly salient. Caregiver-reported valuations tend to underestimate the economic burden of care when compared to institutional data, and do not account for systemic health system savings such as delayed institutionalization. Third, the economic benefits of reduced caregiver time were inferred from cost proxies rather than directly observed through time-use data, potentially introducing measurement bias. Fourth, methodological variability across studies—including differences in outcome measures, time horizons, and costing perspectives—limits the generalizability of findings. Furthermore, regional averages may obscure significant intra-country disparities, which are especially relevant in nations where healthcare access and informal care reliance vary widely by geography and income. Lastly, while several interventions showed favorable ICERs and societal savings, implementation costs (e.g., for training, digital platforms, and clinical supervision) were not fully included, and may impact scalability in real-world contexts. These limitations highlight the need for harmonized multidimensional evaluation frameworks that combine clinical, psychological, and economic metrics to inform evidence-based policy and service design [55].

### 5.2. Implications for Clinical Practice and Policy Planning

An integrated view of clinical and economic perspectives could benefit both practice and policy in the field of dementia care. Firstly, there is a clear need to integrate validated outcomes, in terms of effectiveness and from a comparability perspective. Clinical studies and service evaluations should routinely include both psychometric measures and economic indicators such as QALYs and ICERs in order to comprehensively assess the multidimensional impact of interventions. 

Secondly, the growing evidence base, particularly from programs such as START, reinforces the critical role of clinical psychology and psychometrics. Interventions led by qualified psychologists have demonstrated not only clinical efficacy but also cost-effectiveness, and should, therefore, be prioritized in decisions to commission and fund services. Thirdly, customization of the delivery of interventions should become a standard design principle. Programs should be tailored to the characteristics of caregivers (e.g., age, relationship with the care recipient, employment status) and contextual conditions (e.g., rural or urban access, digital literacy), which are known to influence both outcomes and cost-effectiveness. Constructs such as the WTP and WTA, with the due limitations of a methodological scope, should be considered in the implementation of services in the territory, for better adherence and sustainability as well as to provide insights which are also useful in clinical trials. Moreover, caregiver support policy should reflect the reality that informal caregiving time constitutes a significant indirect healthcare cost. This burden deserves formal recognition in health–economic models, and policy discussions should include the consideration of compensation schemes or service credits. In terms of implementation, scalability and sustainability can be enhanced through low-cost, yet clinically useful interventions, such as unsupervised online CBT, which is particularly well-suited for resource-limited settings when properly adapted. Finally, the combined use of bottom–up (subjective, caregiver-reported, individual, psychological) and top–down (institutional, administrative) data is essential. Only through such integrative approaches can policymakers and researchers construct more robust, multidimensional economic models that reflect both individual experience and social system-level cost dynamics.

## Figures and Tables

**Table 1:** Search query used for systematic search on the Scopus database.

Search Query
(TITLE-ABS-KEY (“caregiver burden”) AND TITLE-ABS-KEY (“cost effectiveness”) OR TITLE-ABS-KEY (cost–effectiveness) OR TITLE-ABS-KEY (“cost utility”) OR TITLE-ABS-KEY (cost–utility) OR TITLE-ABS-KEY (“cost benefit”) OR TITLE-ABS-KEY (cost–benefit) OR TITLE-ABS-KEY (willingness) AND TITLE-ABS-KEY (dementia) OR TITLE-ABS-KEY (ag*ng))

**Table 2:** Scores and judgment for the quality assessment of the included articles.

#	Study Author(s)	Q1	Q2	Q3	Q4	Q5	Q6	Q7	Q8	Score	Quality Judgment
1	[36]	0.5	0	0.5	1	1	1	1	1	6	High
2	[37]	1	1	1	1	1	1	1	1	8	Very high
3	[38]	1	1	1	1	1	1	1	1	8	Very high
4	[39]	1	1	1	1	1	1	1	1	8	Very high
5	[40]	1	1	1	1	1	1	1	1	8	Very high
6	[41]	1	1	1	1	1	1	1	1	8	Very high
7	[42]	1	1	1	1	1	1	1	1	8	Very high
8	[43]	1	1	1	1	1	1	1	1	8	Very high
9	[44]	1	1	1	1	1	1	1	1	8	Very high
10	[45]	0.5	1	1	1	0.5	1	1	1	7	Very high
11	[46]	1	1	1	1	1	1	1	1	8	Very High
12	[47]	1	1	1	1	1	1	1	1	8	Very high
13	[48]	1	1	1	1	1	1	1	1	8	Very High
14	[49]	0.5	1	1	1	0.5	1	1	1	7	Very high

## Data Availability

No new data were created or analyzed in this study.

## Supplementary Materials

The following supporting information can be downloaded at https://www.mdpi.com/article/10.3390/ijerph23010104/s1. Supplementary File S1: Table S1: Summary of non-pharmacological interventions for caregivers of individuals with dementia; Supplementary File S2: Detailed description of indices used across the present study; Supplementary File S3: PRISMA 2020 Checklist [56].

## Appendix A

**Table A1:** Summary of interventions targeting informal caregivers that incorporate both clinical effectiveness assessments and economic evaluations based on stated preference methods.

#ID	Authors	Category of Interv.	Descript. of Interv.	Goals of Interv.	sample Size	Nr. Operators	Duration of Interv.	Cost of Interv.	Intervention Effectiveness	WTP	WTA
1	[43]	Cognition-oriented therapy	CR for early-stage dementia [10 sessions over 3 months + 4 maintenance sessions over 6 months]	To support individuals with dementia in collaboration with caregivers	208 dyads (patient and carer)	Nine occupational therapists and one nurse	9 months (3-month intervention with 6-month follow-up).	9-month costs avg per dyads: GBP3998 (EUR6112)	Improvement with large effect sizes post-intervention and post maintenance (BGSI, DEMQOL-Q, GSES scores)	WTP ≥ GBP2500 (EUR3656) per unit gain in goal attainment	[non-specified]
	[43]	TAU [not specified]	TAU [non-specified]		218 dyads (patient and carer)			9-month costs avg per dyads: GBP4556 (EUR6970)		[non-specified]	[non-specified]
2	[39]	Psychosocial	STrAtegies for RelaTives (START): psychological training program for family carers of dementia patients	To improve carers' mental health, reduce depression and anxiety, and enhance QoL	260 dyads (patient and carer)	Ten graduates in psychology (under supervision) working with 11–32 carers. Eight sessions each	8 sessions across 4 months, follow-up at 4, 8, 12 and 24 months	GBP23,215 (≈EUR38,418) per carer	carers’ depression/anxiety reduction and improved QoL short- and long-term.	Decisional Thresholds between GBP20,000 (EUR30.888) to GBP30,000 (EUR46.332)/QALY	[non-specified]
	[39]	TAU [not specified]	TAU [non-specified]	[non-specified]	87 dyads (patient and carer)	[non-specified]		[non-specified]		[non-specified]	[non-specified]
3	[40]	Psychosocial	STrAtegies for RelaTives (START) in England.	To improve mental health and QoL for family carers; reduce health and social care costs	48,684 carers (Scalability projections 2025)	3087 (2025)	8 sessions across 4 months, follow-up at 4, 8, 12 and 24 months	GBP236 per carer [EUR27,848] (2025)	Significant improvements in depression and anxiety levels among carers.	Decisional Thresholds between GBP20,000 (EUR30.888) to GBP30,000 (EUR46.332)/QALY	[non-specified]
	[40]				55,333 carers (Scalability projections 2030)	3523 (2030)		GBP260 per carer [EUR30,680] (2030)			
	[40]				60,721 carers (Scalability projections 2035)	3854 (2035)		GBP288 per carer [EUR33,984] (2035)			
	[40]				62,752 carers (Scalability projections 2040)	3978 (2040)		GBP316 per carer [EUR37,336] (2040)			
4	[37]	Technological Support	Assistive Technology and Telecare (ATT) to manage risks and maintain independence for people with dementia	To extend time living independently, reduce safety risks, and support caregiver well-being.	495 dyads (patient and carer) [247 received a standard attention through an average of 6.5 telephone sessions (about 54 min each)]	Teams included an occupational therapist, a nurse, and a psychologist	12 weeks, with a mean of 8.4 sessions per dyad with ongoing assessments.	GBP63,554 per dyad (EUR75,502)	No significant improvement in QoL; minor reductions in caregiver burden.	The study implicitly adopts the NICE range (20,000 (EUR30.888) to GBP30,000 (EUR46.332)/QALY)	Not applicable in this context
	[37]	TAU [not specified]	TAU [non-specified]	[non-specified]	247 dyads (patient and carer)	[non-specified]		GBP67,647 per dyad (EUR80,365)	[non-specified]		
5	[38]	Technological Support	Online cCBT with telephone in dementia informal carers	To improve caregiver mental health (reduce depression/anxiety), prevent “caseness” on GHQ-12, and increase quality-adjusted life years (QALYs)	176 carers	Trained staff [number non-specified]	6 months of intervention	GBP468 per caregiver (EUR724)	Significant reduction in anxiety and depression; minor improvements in caregiver QOL.	WTP = GBP19,500/QALY (EUR23,166)	[non-specified]
										WTP = GBP12,900/GHQ-12 case prevented (EUR15,325)	
	[38]		Online cCBT without telephone support in dementia informal carers					GBP458 per caregiver (EUR708)	Limited reduction in anxiety and depression; minor improvements in caregiver QOL.	WTP = GBP27,600/QALY (EUR32,789)	[non-specified]
	[38]		Psychoeducation in dementia informal carers					GBP358 per caregiver (EUR554)	[non-specified]	[non-specified]	
6	[44]	Cost–effectiveness study	Apply literature-based WTP values to assess the cost–effectiveness of the Tailored Activity Program (TAP)	To reduce burden	60 dyads (patient and carer)	One occupational therapist per dyad	in-home, 8-session (3-month) program	[non-specified]	TAP reduced “on duty” caregiving by an average of 6.9 h/day and “doing things” by 3.3 h/day; also reduced behavioral occurrences and increased activity engagement	“On duty”: USD1.06–USD4.58/hour (≈ EUR1.17–EUR5.06/hour) – “Doing things”: USD2.21–USD9.57/hour (≈ EUR2.44–EUR10.57/hour)	[non-specified]
7	[41]	Contingent-valuation WTP experiment	Tailored in-home program	Equip informal caregivers to manage behavioral symptoms, reduce burden, and improve patient engagement	260 dyads (patient and carer)	One healthcare professional per dyad	8-session (3-month)	[non-specified]	[non-specified]	WTP = USD288.00 (≈ EUR 318.25)	[non-specified]
8	[42]	Contingent-valuation WTP experiment	Tailored in-home program	To determine WTP value per session for the program	223 dyads (patient and carer)	One healthcare professional per dyad	8-session (3-month)	USD941per dyad for 4 months (≈EUR 1039 in 2025)	[non-specified]	WTP = USD288.00 (≈ EUR 318.25)	[non-specified]
9	[47]	CMV experiment	[non-specified]	To estimate WTP/WTA for leisure time lost due to informal caregiving and quantify the aggregate annual economic burden of that lost leisure	315 carers	[non-specified]	[non-specified]	[non-specified]	[non-specified]	WTP = 99,200 KRW/hour (~USD7.74; ~EUR7.12)	WTA = 2848.4 KRW/hour (USD10.82; ≈ EUR9.95)
10	[48]	Cross-sectional CVM study	Three hypothetical patient treatments designed to reduce caregiver burden	To reduce burden from moderate to low	109 carers	[non-specified]	[non-specified]	[non-specified]	Reduction in burden from moderate to low (no measured clinical outcomes)	WTP= 2200 CHF per year (≈EUR 2208)	[non-specified]
11	[49]	CMV experiment	Three hypothetical, out-of-pocket treatments (stabilization, cure, no-burden) presented in a contingent-valuation survey	To elicit patients’ and caregivers’ WTP for each treatment; test for mutual versus unilateral altruism in these WTP values	126 dyads (patient and carer)	[non-specified]	[non-specified]	Payment-card bids ranged from 5000 to 500,000 CHF (≈5000–500,000 EUR)	[non-specified]	TP calculated as maximum percentage of caregiver wealth willing to pay. Stabilization: WTP USD23.9 (EUR28.8), Cure: WTP USD30.7 (EUR37), No burden: WTP 17.5 (EUR21.1)	[non-specified]
12	[50]	CMV experiment	Measure informal caregivers’ max WTP for a 1-hour/week reduction, or min WTA for a 1-hour/week increase, in their least-preferred care task	To estimate the economic value of informal caregiving by eliciting WTP and WTA per hour of caregiving a	371 carers	[non-specified]	[non-specified]	[non-specified]	[non-specified]	WTP = 25.31 CNY/hour (≈ EUR 3.29) per hour per week	WTA = 38.66 CNY/hour (5.83 EUR/hour)
13	[36]	DCE experiment	Hypothetical bundles of public home care services to elicit preferences for dementia home support	To quantify caregivers’ Willingness-to-Pay for home care service attributes to guide dementia-care service design and policy in Milan.	93 carers	[non-specified]	[non-specified]	Not a real intervention; cost attribute levels in the choice sets were monthly co-payments of EUR130, EUR260 or EUR390	Significant positive utility for increased hours, mixed health and social care, and professional peer support groups	WTP= EUR5.13 (for additional home care hour per month) WTP= EUR290 (for mixed health and social care) WTP= EUR171 (for caregiver support groups with professional facilitation)	[non-specified]
14	[46]	model-based health–economic evaluation—specifically, a Markov decision-analytic cost-effectiveness analysis	Markov Model, from the healthcare sector perspective. (parameters from the dementia care literature).	To run cost–utility analysis of TAP vs. usual care, for the person with dementia and their caregiver	1000 per dyad [patients starting in the mild state (cycle 0) and followed until death]	One therapist per dyad	Up to eight sessions (home visits and telephone calls) over four months or more	USD 1200 per dyad per cicle(≈EUR 672)	[non-specified]	WTP = USD 50,000 (EUR 28,000) calculated on Hypothetical Australian social care thresold	[non-specified]

**Table A2:** Summary of interventions targeting informal caregivers that incorporate both clinical effectiveness assessments and economic evaluations based on stated preference methods and Stated Preference Methods Experiments. The items are classified by category of intervention/ experiment: BGSI (Brief Geriatric Screening Instrument); GSES (General Self-Efficacy Scale); DEMQOL-U (Dementia Quality of Life–Utility); CBT (cognitive-behavioral therapy); NHS (National Health Service–UK); cCBT = computerized cognitive–behavioral therapy; GHQ-12 = General Health Questionnaire-12; societal perspective costs = valuation of unpaid caregiver time; health and social care perspective cost = valuation of public expenditure; TAU: Treatment As Usual; CR: Cognitive Rehabilitation; DEMQOL-U: Dementia Quality of Life Health-Utility Measure; EQ-5D-3L: EuroQol-5 Dimensions, 3-Level Version; WTP (Willingness-to-Pay); WTA (Willingness-to-Accept). ICER is calculated using values other than QALY (e.g., points of goal attainment) and using QALY values; societal weights in QALY: modifications to the basic QALY calculation that account for societal preferences beyond individual utility.

#ID	Authors	Category of Intervention	ICER * (Calculated Using Values Other than QALY)	Cost Utility (Cost per QALY) * ICER Calculated Using QALy	QALY Gains	Instruments for QALY Evaluation (Quality-Adjusted Life Years)	WTP (Willingness-to-Pay)	WTA (Willingness-to-Accept)	DALY (Disability-Adjusted Life Years)	Sensitivity Analysis Results
1	[43]	Cognition-oriented therapy	(1) Patient (vs. TAU) -*Goal Attainment, +1.32 BSGI points* -Societal perspective (incl. unpaid caregiver time): −GBP9 per 1.32-point gain (≈−EUR10) *(2) Self-efficacy* -Self-efficacy, +1.53 GSES points -Societal perspective (incl. unpaid caregiver time): −GBP2961 per 1.53-point gain (≈−EUR3268)	Societal perspective: −GBP1052,000/QALY (≈−EUR1716,200/QALY) (incl. unpaid caregiver time)	No evidence of cost–effectiveness	[non-specified]	CR cost–effective from health+social care and societal perspectives: WTP ≥ GBP2500 (EUR3656) per unit gain in goal attainment *	[non-specified]	[non-calculated]	Robust results; validated across multiple sensitivity scenarios with imputed data
			[non-specified]	−GBP902,000/QALY (≈−EUR1,472,900/QALY) (incl. unpaid caregiver time)		EQ-5D by applying societal weights				
2	[39]	Psycosocial	Per HADS-T point (8 m = 2.10): GBP118/point (≈EUR18,362)	Per QALY (8 m): GBP6000/QALY (≈EUR9336.60) mean incremental health and social care cost for START vs. TAU over 8 months: GBP252 (−GBP28 to GBP565) (≈EUR388);	0.03 at 8 months;	EQ-5D by applying societal weights *	Thresholds between GBP20,000 (EUR30.888) to GBP30,000 (EUR46.332) per QALY (WTP was based on decision-makers' hypothetical valuations of the monetary worth of a unit gain in goal attainment (perhaps following NICE guidelines)	Not monetarily specified in the document.	[non-calculated]	Robust in sensitivity analyses across cost and QALY estimate variations.
3	[40]	Psycosocial	[non-calculated]	2025: −GBP43,900/QALY (≈−EUR52,200)	QALY gain per person (24 months) 0.03	from EQ-5D utilities for carers.	Thresholds of GBP20,000 (EUR30.888) to GBP30,000 (EUR46.332) per QALY	Not monetarily specified in the document.	[non-calculated]	Robust in sensitivity analyses across cost and QALY estimate variations.
	[40]			2030: −GBP47,000/QALY (≈−EUR55,800)						
	[40]			2035: −GBP50,100/QALY (≈−EUR59,500)						
				2040: −GBP55,900/QALY (≈−EUR66,400)						
4	[37]	Technological Support	Institutionalization-free days at 104 weeks Health and social care perspective: –GBP115 (EUR171 iEUR) per additional day	Institutionalization-free days at 104 weeks Societal perspective: EQ-5D proxy reported (by Carer): −GBP120,125/QALY (−EUR166,969)	Minimal QALY gains; participant ratings lower than controls.		Willingness-to-Pay thresholds not specifically addressed in analysis. The study implicitly adopts the NICE range (GBP20–30 k/QALY) as its reference	Not applicable in this context	[non-calculated]	Robust in sensitivity analyses across cost and QALY estimate variations.
5	[38]	Technological Support	*GHQ-12 (per 1-point improvement:* cCBT vs. Psychoeducation: GBP80/point → (102/point) cCBT+Telephone vs. Psychoeducation: GBP100/point (EUR127/point)	cCBT vs. sychoeducation: GBP8130/QALY (≈EUR10,335/QALY	Telephone-supported CBT: QALY gain 0.003.	By mapping SF-12 responses to SF-6D utilities and integrating these utilities over 26 weeks	For prevention of a GHQ-12 “case” GBP12,900 (EUR16.600) per case prevented *	[non-calculated]	[non-calculated]	Robust results with sensitivity analysis; outcomes consistent across multiple scenarios.
			GHQ-12 “case” prevented: cCBT+Telephone vs. Psychoeducation: GBP610 per case (EUR775 per case)	cCBT vs. Psychoeducation: GBP8130/QALY (EUR10,335/QALY) cCBT+Telephone vs. Psychoeducation: GBP39,680/QALY (EUR50,440/QALY)	Unsupported CBT: QALY gain 0.01					
6	[44]	Cost–effectiveness study	-Cost to reduce 1 h “on duty”: $1.10 (≈EUR1.21) -Cost to reduce 1 h “doing things”: $2.37 (≈EUR2.62)	[non-specified]	[non-specified]	[non-specified]	-“On duty”: USD1.06–USD4.58/hour (≈EUR1.17–EUR5.06/hour) -“Doing things”: USD2.21–USD9.57/hour (≈EUR2.44–EUR10.57/hour)	[non-specified]	[non-specified]	– 50% cost–effective at WTP ≥ USD1.20/h (≈EUR1.33) for “on duty” and ≥ $USD.40/h (≈EUR2.65) for “doing things”
7	[41]	Contingent-valuation WTP experiment	[non-specified]	[non-specified]	[non-specified]	[non-specified]	Mean unadjusted WTP (baseline): USD36.54 per session (SD 83.56) (≈ EUR 40.32) Mean adjusted WTP: USD36.00 per session (95% CI USD26.73–USD45.27) (≈ EUR 39.74) Equivalent for full 8-session program: USD292.32 (SD 668.48) (≈ EUR 322.94) unadjusted, or USD288.00 (95% CI USD213.82–USD362.18) (≈EUR 318.25) adjusted	[non-specified]	[non-specified]	No formal sensitivity analyses reported
8	[42]	Contingent-valuation WTP experiment	[non-specified]	[non-specified]	[non-specified]	[non-specified]	Mean adjusted WTP: USD36.00 per session (95% CI USD26.72–USD45.27) (≈EUR 39.74/session) Extrapolated for the full 8-session program: USD288.00 (95% CI USD213.82–USD362.18) (≈EUR 318.25 total)	[non-specified]	[non-specified]	No formal sensitivity analyses reported
9	[47]	CMV experiment	[non-specified]	[non-specified]	[non-specified]	[non-specified]	9200 KRW/hour (USD7.74; ≈EUR7.12) for all respondents without reference price; trimmed mean for regular caregivers 9312.5 KRW/hour (USD7.84; ≈EUR7.21)	2848.4 KRW/hour (USD10.82; ≈EUR9.95) without reference price; trimmed mean for regular caregivers 12,739.4 KRW/hour (USD10.72; ≈EUR9.87)	[non-specified]	Trimmed mean (drop top/bottom 10%) to limit extreme WTP/WTA bias; WTA > WTP (t = −8.06, *p* < 0.01).
10	[48]	Cross-sectional CVM study	[non-specified]	[non-specified]	[non-specified]	[non-specified]	2200 CHF per year (≈EUR 2208)	[non-specified]	[non-specified]	[non-specified]
11	[49]	CMV experiment	[non-specified]	[non-specified]	[non-specified]	[non-specified]	WTP framed as a one-time payment Expressed as percent of household wealth (absolute CHF amounts not reported): Patients: Stabilization 13.8%, Cure 21.6%, No burden 21.9% Caregivers: Stabilization 23.9%, Cure 30.7%, No burden 17.5%	[non-specified]	[non-specified]	T-tests: Patients’ WTP for Stabilization < Cure and No burden; caregivers’ WTP ranks Cure > Stabilization > No burden; no patient–caregiver difference for No burden.
12	[50]	CMV experiment	[non-specified]	[non-specified]	[non-specified]	[non-specified]	Valuation framed per hour per week; mean WTP 25.31 CNY/hour (≈EUR 3.29)	38.66 CNY/hour (5.83 EUR/hour)	[non-specified]	No formal sensitivity analysis reported
13	[36]	DCE experiment	[non-specified]	[non-specified]	[non-specified]	[non-specified]	EUR5.13 per additional home care hour per month (marginal WTP) EUR290 for mixed health and social care EUR171 for caregiver support groups with professional facilitation	[non-specified]	[non-specified]	No formal sensitivity analysis reported
14	[46]	Model-based health–economic evaluation—specifically, a Markov decision-analytic cost–effectiveness analysis	[non-specified]	Females: AUD 8445 (EUR 4728) per QALY gained Males: AUD 9371 (EUR 5247) per QALY gained	Females: 0.89 QALYs Males: 0.83 QALYs	[non-specified]	AUD 50,000 (EUR 28,000) calculated on hypothetical Australian social care threshold	[non-specified]	[non-specified]	Sensitivity analysis conducted

* NICE-aligned.
