# Supplementary material for: A Review of Dementia Caregiver Interventions: Valuing Psychological Well-Being and Economic Impact Through the State-Preference Method
Source: Int J Environ Res Public Health. 2026 Jan 12;23(1):104. doi: 10.3390/ijerph23010104 (PMC12840879; doi:10.3390/ijerph23010104)
Supplement: Supplementary file 1 [file ijerph-23-00104-s001.zip › ijerph-4052642-Supplementary File S1.pdf]

Supplementary Materials S1

Table S1. Summary of non-pharmacological interventions for caregivers of individuals with dementia, adapted from Huo et al. (2021).

The table includes descriptions, goals, personnel involved, costs (converted to 2025 EUR values using an exchange rate of USD 1 = EUR 0.9670 and a cumulative inflation adjustment of 13.3% BBC), effectiveness, and cost-effectiveness indicators such as ICER and WTP. Interventions are categorized according to their type, duration, and reported utility gains QALYs.

Intervention Type	Description of intervention	Goals of intervention	Number of operators involved	*Cost of intervention	Costs per patient and caregiver	Effectiveness	Cost-effectiveness	ICER	Cost Utility	Duration	WTP (Willingness to Pay)	WTA (Willingness to Accept)	QALY Gains	Cost per QALY	DALY	Sensitivity Analysis
**Psychosocial interventions**	Provide emotional support and coping strategies for caregivers.	Reduce stress and emotional burden of caregivers.	Typically 1-2 counselors or therapists per caregiver.	EUR 1,081.08 – EUR 3,243.24 per caregiver annually.	Variable; includes emotional costs and reduced productivity.	Improves mental health (e.g., anxiety, depression).	Generally cost-effective when caregiver burden is reduced.	EUR 10,956.11 – EUR 21,912.22 per QALY.	Improves caregiver and patient well-being.	6-12 months.	EUR 2,739.03 per program.	Not explicitly reported.	0.1 - 0.3 QALY annually.	EUR 10,956.11 – EUR 21,912.22.	Not explicitly reported.	Consistent across caregiver demographics.
**Dementia management**	Educational programs to help caregivers manage behavioral and psychological symptoms of dementia.	Improve caregiver skills in managing dementia-related challenges.	1-2 educators or trainers per group.	EUR 1,081.08 – EUR 3,243.24 per caregiver annually.	Dependent on educational program duration and frequency.	Increases caregiver knowledge and preparedness.	Highly cost-effective in reducing hospitalizations and crises.	EUR 16,434.17 per QALY gained.	Reduces healthcare utilization and caregiver stress.	3-6 months.	EUR 3,286.83 annually.	Not explicitly reported.	0.15 - 0.25 QALY annually.	EUR 16,434.17.	Not explicitly reported.	Highly robust; no major variability in results.

**Home support programs**	Home care assistance combined with caregiver training and support.	Enhance caregiving efficiency and reduce caregiver strain.	Variable; depends on the intensity of home care support required.	EUR 2,191.22 to EUR 8,764.89 per year depending on support level.	Significant reduction in stress and improved productivity.	Reduces emotional strain and improves patient outcomes.	Cost-effective in high-burden cases.	Not explicitly reported; likely <EUR 27,390.27 per QALY.	Improves overall efficiency in caregiving and health outcomes.	Continuous or as needed.	EUR 5,478.05 annually.	Not explicitly reported.	0.2 - 0.4 QALY annually.	Not explicitly calculated.	Not explicitly reported.	Confirmed effective in high-burden scenarios.
**Respite care interventions**	Temporary relief for caregivers through replacement caregiving services.	Provide temporary relief and improve caregiver well-being.	1-3 professionals for temporary caregiving shifts.	EUR 1,095.61 to EUR 4,382.44 per episode.	Temporary relief reduces caregiver fatigue; costs vary.	Reduces fatigue and improves caregiver health.	Effective in reducing long-term healthcare costs.	Variable; depends on frequency and caregiver demographics.	Provides temporary improvement in caregiver health metrics.	Variable; typically 1-2 weeks per episode.	EUR 2,191.22 per episode.	Not explicitly reported.	0.05 - 0.15 QALY annually.	Variable, <EUR 27,390.27	Not explicitly reported.	Variable results based on caregiver demographics.
**Activity-based interventions**	Engage patients in meaningful activities to improve their quality of life and reduce caregiver burden.	Improve patient engagement and quality of life; reduce caregiver stress.	1 therapist per caregiver-patient pair.	EUR 1,314.73 per cycle (4 months).	Moderate reductions in caregiver burden and associated costs.	Enhances quality of life and engagement for both parties.	Moderately cost-effective in improving QALYs.	Ranges EUR 10,956.11 - EUR 32,868.33 per QALY.	Improves quality of life and reduces burden.	4-month cycles.	EUR 1,643.42 per cycle.	Not explicitly reported.	0.1 QALY per cycle.	EUR 10,956.11 - EUR 32,868.33.	Not explicitly reported.	Stable across scenarios; minor variations.
**Group therapies**	Group sessions for caregivers to share experiences and management strategies.	Build community among caregivers and improve collective coping mechanisms.	1 group facilitator per session.	EUR 547.81 to EUR 1,643.42 per group session cycle.	Reduces emotional burden; minor financial implications.	Provides emotional support and knowledge sharing.	Variable; depends on group size and frequency.	Not calculated; assumed <EUR 16,434.17 per QALY.	Community benefits through shared knowledge.	6-week sessions.	EUR 1,095.61 per group cycle.	Not explicitly reported.	Not reported explicitly.	Not explicitly calculated.	Not explicitly reported.	Minimal impact of demographic variations.

**Technological support**	Use of apps, online platforms, or devices to provide remote assistance or educational resources.	Enhance accessibility to resources and training.	None directly; remote support via technology.	EUR 54.78 to EUR 328.68 per caregiver	Minimal cost; dependent on technological solution.	Improves accessibility to resources and caregiver skills.	Highly cost-effective due to scalability.	Minimal cost; not applicable.	Extends reach to remote or underserved caregivers.	Continuous, based on usage.	EUR 328.68 annually.	Not explicitly reported.	Not reported explicitly.	Not explicitly calculated.	Not explicitly reported.	High consistency across user groups.
