# Supplementary material for: A Review of Dementia Caregiver Interventions: Valuing Psychological Well-Being and Economic Impact Through the State-Preference Method
Source: Int J Environ Res Public Health. 2026 Jan 12;23(1):104. doi: 10.3390/ijerph23010104 (PMC12840879; doi:10.3390/ijerph23010104)
Supplement: Supplementary file 1 [file ijerph-23-00104-s001.zip › ijerph-4052642-Supplementary File S2.pdf]

**Supplementary materials S2**

S2. Detailed description of indices used across the present study.

## Definition and Calculation of Quality-Adjusted Life Years (QALYs)

QALYs is a measure of health outcomes that combines the length of life with its quality. One QALY represents one year of perfect health. As a universally applicable outcome measure, QALYs enable strategic decision-making in health systems, for example, in allocating budgets across clinical specialties or prioritizing within national healthcare frameworks (Knapp et al., 2020).

$$\text{QALYs} = \sum_{t=1}^{T} (Ut \times \Delta t)$$

Where

$U_t$ = utility value (quality of life) of the caregiver at time t (range 0 to 1)

$\Delta_t$ = time duration (e.g., 1 year or fractions thereof)

T = total time horizon of caregiving (e.g., number of years)

## Definition and Calculation of Disability-Adjusted Life Year (DALY)

Disability-Adjusted Life Year (DALY), a composite measure of health loss that quantifies the burden of disease by summing years of life lost due to premature death (YLL) and years lived with disability (YLD). It reflects the total number of years lost due to ill-health, disability, or early death (Murray and Lopez, 1996; WHO, 2020).

$$DALY = YLL + YLD$$

Where:

YLL (Years of Life Lost) = N×LN×L

*(N: number of deaths, L: standard life expectancy at age of death)*

YLD (Years Lived with Disability) = I×DW×LI×DW×L

*(I: number of incident cases, DW: disability weight, L: duration of disability)*

Unlike Cost–Effectiveness and Cost–Utility approaches, Cost–Benefit Analysis (CBA) translates both the costs and the benefits of an intervention into monetary terms, allowing for a direct economic comparison.

**Cost–Effectiveness Analysis in Dementia Care: Key Metrics and Approaches**

Cost–Effectiveness Analysis (CEA) approach compares the costs of different interventions to their effectiveness in achieving specific health outcomes, such as delaying institutionalization or improving caregiver well-being. Outcomes are typically measured in natural units (Chong, 2003) such as years of life gained or QALYs. To accurately assess the economic value of interventions targeting dementia care and informal caregiving, it is essential to adopt standardized models and key economic indicators. Among the most widely used tools in health economics are the Incremental Cost–Effectiveness Ratio (ICER), the Incremental Cost–Utility Ratio (ICUR), and various Cost–Benefit Analysis (CBA) metrics. These functions allow for a comparative evaluation of costs and outcomes across different care strategies, providing critical insights for healthcare decision-making and policy development.

1. *The Incremental Cost–Effectiveness Ratio (ICER)*

A fundamental metric within Cost–Effectiveness Analysis is the Incremental Cost–Effectiveness Ratio (ICER), which enables the comparison of two or more interventions by relating their differences in cost to differences in health outcomes. The ICER provides a standardized measure for evaluating whether the additional cost of an intervention is justified by the additional benefit it produces (Drummond et al.2015).

$$\text{ICER} = Cost_{intervention} - Cost_{control}/Effect\_intervention - Effect\_control$$

Where
Cost_intervention: Total cost of the intervention (e.g., care program).

Cost_control: Total cost of the standard or control treatment.

Effect_intervention: Health outcome of the intervention, typically in QALYs.

Effect_control: Health outcome of the control treatment, typically in QALYs.

A lower ICER indicates greater Cost–Effectiveness. If the ICER is below a certain threshold, (e.g., USD 50,000/QALY, where the cost amount refers to the full intervention), the intervention is generally considered cost-effective. More in detail, as cost estimates for different types of care activities vary significantly depending on the nature of the tasks performed (Engel et al., 2021), it has been introduced—as will be discussed in more detail later—the possibility of deriving a monetary value (e.g., governments, insurance agencies) use cost per QALY to compare different health interventions on equal footing (Koopmanschap et al., 2008). In this case, ICER value is compared to the predetermined threshold chosen (often by policy):

$$If\ ICER \leq WTP\ threshold \Rightarrow Intervention\ is\ cost - effective$$

$$If\ ICER > WTP\ threshold \Rightarrow Intervention\ is\ not\ cost - effective$$

2. *Cost–Benefit Analysis (CBA)*

Cost–Benefit Analysis (CBA) facilitates decision-making by determining whether the financial value of the benefits justifies the investment required. The Cost Benefit Analysis method monetizes both the costs and benefits of an intervention, expressing them in a common unit of currency (e.g., USD). This enables a direct comparison of the economic value of the intervention's benefits to its associated costs (Boardman et al., 2018).

$$\text{Net Benefit} = Total\ Benefits - Total\ Costs$$

$$\text{Benefit Cost Ratio (BCR)} = Total\ Benefits\ /\ Total\ Costs$$

Where:

- Total Benefits: The monetary value of all benefits derived from the intervention.

- Total Costs: The monetary value of all costs incurred by the intervention.

A positive Net Benefit or a BCR greater than 1 indicates that the benefits outweigh the costs.

More specifically, the literature outlines that a limited number of studies adopt a social perspective, with CUAs and Markov models representing the most prevalent modeling approach (Huo et al., 2021; Brennan et al., 2006).

3. *Contingent Evaluation Method (CVM)*

The CVM assesses the monetary value placed on informal care by estimating the minimum amount of money an informal caregiver would require in exchange for providing an additional hour of informal care (WTA – Willingness to Accept). Alternatively, it also considers the maximum amount of money the caregiver would be willing to pay for reducing their caregiving responsibilities by one hour (WTP – Willingness to Pay, Hanneman, 1994; van den Berg et al., 2004).

$$Net\ Benefit\ =\ WTP\ -\ Cost$$

Where:

-WTP = Willingness to Pay (or WTA for compensation)

- Cost = Actual cost of intervention or support program

To address the limitations associated with contingent valuation methods, the recent literature has advocated for the application of conjoint analysis, specifically discrete choice experiments, for the valuation of informal care; unlike traditional methods, conjoint analysis does not require respondents to directly state a monetary value (e.g., Quaife et al., 2018; de Bekker-Grob et al., 2019). Instead, it involves presenting respondents with hypothetical scenarios related to informal care and instructing them to make trade-offs between different aspects of these scenarios:

$$U_{\mathrm{ij}}\ =\ V_{\mathrm{ij}}\ +\ \varepsilon_{\mathrm{ij}}$$

Where:

-$U_{ij}$ = total utility of alternative *j* for individual *i*

-$V_{ij}$ = observable (systematic) component of utility

-$\varepsilon_{ij}$ = random error component

4. *Discrete Choice Experiments (DCEs)*

A central concern with stated preference methods like DCEs is the potential for *hypothetical bias*. Since respondents answer hypothetical questions, their expressed preferences may not accurately reflect their actual choices in real-world situations with real economic consequences (Rakotonarivo, et Al. 2016). However, within the context of dementia care, the application of conjoint analysis, along with the well-being method, remains unexplored and needs further investigation (e.g. Engels et al.

2021). In economic evaluations—especially those assessing interventions for caregivers of people with dementia—sensitivity analysis plays a critical role in exploring uncertainty in input parameters, costs, and outcomes (Drummond et al., 2015; Neumann et al., 2017).

**Sensitivity Analyses in Cost–Effectiveness Evaluation: Methods and Applications**

1. *One-Way Sensitivity Analysis*

One-way sensitivity method evaluates how varying a single parameter influences the outcome, such as the incremental cost–effectiveness ratio (ICER). For example, the utility value associated with caregiver quality of life (u) might be varied while keeping other inputs constant (Drummond et al., 2015).

$$ICER = (Cost\_intervention - Cost\_comparator) / (Effect\_intervention - Effect\_comparator)$$

2. *Two-Way and Multi-Way Sensitivity Analysis*

Two-Way (Multi-Way) Sensitivity Analysis assesses the combined impact of simultaneously varying two or more parameters. This is particularly useful when evaluating interactions between caregiver time costs and effectiveness (Gervès-Pinquié et al., 2022).

$$ICER(u_1, c_1) = (C_1(u_1, c_1) - C_0) / (E_1(u_1, c_1) - E_0)$$

3. *Probabilistic Sensitivity Analysis (PSA)*

Probabilistic Sensitivity Analysis (PSA) assigns statistical distributions to input parameters and runs simulations to produce a distribution of ICER values (Neumann et al., 2017):

$$\theta_{\mathrm{i}} \sim Distribution(\mu_{\mathrm{i}}, \sigma_{\mathrm{i}}), \quad i = 1, 2, \ldots, n$$

Each simulation iteration produces:

$$ICER\hat{}(j) = (C_1\hat{}(j) - C_0\hat{}(j)) / (E_1\hat{}(j) - E_0\hat{}(j)), \quad j = 1, \ldots, N$$

This method is strongly recommended by health technology assessment institutions like WHO (World Health Organization, 2020).

4. *Scenario Analysis*

Scenario analysis evaluates the effect of distinct assumptions or conditions:

- Scenario A (excluding informal care):

$$TotalCost\_scenarioA = C\_formal$$

- Scenario B (including informal caregiver time):

$$TotalCost\_scenarioB = C\_formal + C\_informal$$

This is especially useful in caregiver-related evaluations, where informal care contributes substantially to societal costs (Gervès-Pinquié et al., 2022).

5. *Threshold and Structural Sensitivity Analysis*

Threshold Analysis determines the value a parameter must reach for an intervention to be considered cost-effective:

$$ICER = \lambda \quad \Rightarrow \quad \lambda = (C_1 - C_0) / (E_1 - E_0)$$

It is particularly helpful when decision-makers want to identify the maximum acceptable cost or minimum required effect size (Drummond et al., 2015).

Structural Sensitivity Analysis explores how changes in the structure of the economic model affect outcomes. For example, caregiver burden over time may be modeled as follows:

- Linear progression

$$Burden(t) = \alpha \cdot t$$

- Exponential progression

$$Burden(t) = \alpha \cdot e\text{^}(\beta \cdot t)$$

Such structural variations are highly relevant for dementia, where caregiver needs intensify non-linearly as the disease progresses (Neumann et al., 2017).
